# Healthcare consumer acceptability of routine use of the EQ-5D-5L in clinical care: a cross-sectional survey

**DOI:** 10.1007/s11136-024-03598-z

**Published:** 2024-02-07

**Authors:** David A. Snowdon, Taya A. Collyer, Lucy Marsh, Velandai Srikanth, Richard Beare, Stephanie Baber, Kim Naude, Nadine E. Andrew

**Affiliations:** 1 National Centre for Healthy Ageing, 2 Hastings Road, Frankston, VIC Australia; 2 Academic Unit, Peninsula Health, Frankston, VIC Australia; 3 Peninsula Clinical School, Central Clinical School, Monash University, Melbourne, VIC Australia; 4 Developmental Imaging, Murdoch Children’s Research Institute, Melbourne, Australia; 5 Physiotherapy Department, Golf Links Road Rehabilitation Centre, Peninsula Health, Frankston, VIC Australia

**Keywords:** Patient reported outcome measures, Quality of life, Outcome assessment, Quality of health care, EQ-5D-5L

## Abstract

**Purpose:**

Patient reported outcome measures, such as the EQ-5D-5L, provide a measure of self-perceived health status or health-related quality of life. Understanding the consumer acceptability of a patient reported outcome measure can help to decide about its implementation across a healthcare organisation and possibly increase the likelihood of its use in clinical care. This study established the acceptability of the EQ-5D-5L from the perspective of clients receiving healthcare, and determined if acceptability varied by client sub-types.

**Methods:**

A cross-sectional survey explored clients’ experience of the EQ-5D-5L. Eligible clients were aged ≥ 18 years and completed the EQ-5D-5L on admission and discharge to one of two multi-disciplinary community health services. Likert scale items explored acceptability, and open-ended questions determined if the EQ-5D-5L reflects experience of illness. Associations between acceptability and client characteristics were established using χ^2^ test. Open-ended questions were analysed using content analysis.

**Results:**

Most of the 304 clients (mean age 70 years, SD 16) agreed that the EQ-5D-5L: was easy to use/understand (n = 301, 99%) and useful (n = 289, 95%); improved communication with their therapist (n = 275, 90%); and made them feel more in control of their health (n = 276, 91%). Most clients also agreed that they wished to continue using the EQ-5D-5L (n = 285, 93%). Clients aged ≥ 60 years reported lower acceptability. Clients noted that the EQ-5D-5L did not capture experience of illness related to fatigue, balance/falls, cognition, and sleep.

**Conclusion:**

The EQ-5D-5L is acceptable for use in care but does not capture all aspects of health relevant to clients, and acceptability varies by subgroup.

**Supplementary Information:**

The online version contains supplementary material available at 10.1007/s11136-024-03598-z.

## Plain English summary

Patient reported outcome measures, or PROMs, are questionnaires completed by patients about their own health and well-being. Information from PROMs can help patients to receive a high quality of care. However, routine collection of PROMs across a healthcare organisation is difficult, and requires both consumer and clinician input to be successful. In this study, we explored consumers’ experience of using the EQ-5D-5L, and assessed whether the instrument was relevant to their experience of illness. The results indicate that the EQ-5D-5L is acceptable to most consumers, however some may require an additional PROM to ensure their experience of illness is captured. Specifically, consumers identified that the EQ-5D-5L did not ask about fatigue, balance/falls, cognition, and sleep, but generally covered most aspects of health that were important to consumers. Findings from this study mean that the EQ-5D-5L is appropriate, and could potentially be used successfully across a healthcare organisation. They also indicate that the EQ-5D-5L may be complemented with additional PROMs, or questions, that capture the aspects of health that were missing such as cognition and fatigue.

## Background

Globally, the number of people aged 60 years and over has tripled since 1950 and is projected to double over the next 30 years [1]. With health disorders in those aged 60 years and over accounting for 23% of the total global burden of disease, and 50% in high income countries, an ageing population will result in greater demand for healthcare [2]. For example, the population of older Australians has doubled since 1995 from 2.1 million to 4.2 million, which has seen an increase in healthcare costs from $39 billion to $203 billion dollars [3, 4]. An ageing population and growing demand for healthcare, means that healthcare organisations face the challenge of providing services that improve outcomes that are meaningful to consumers at the lowest possible cost [1, 5].

Routinely collected clinical data in electronic health records are considered a key resource for informing organisations on how they can transform healthcare to better meet the needs of consumers [6]. However, patient reported data is underrepresented in routinely collected clinical data. This can lead to underestimates of the impact of disease on important aspects of health such as quality of life and overestimates of the effectiveness of healthcare interventions [6, 7]. As such, there is a need to incorporate consumers’ perspectives on their health into routinely collected clinical datasets [8].

Patient reported outcome measures (PROMs) provide a measure of self-perceived health status or health-related quality of life [9] which can be used secondarily to determine the effectiveness of healthcare interventions via the impact on outcomes that are meaningful to the consumer. Embedding PROMs into routinely collected clinical data to inform provider-patient decisions, healthcare interventions and value-based healthcare models, has the potential to transform healthcare to better meet the needs of consumers [10–14]. PROMs can be classified as generic, or specific to a disease or health condition [15–17]. Because generic PROMs are applicable to consumers with any disease or health condition, they are appropriate for routine use across a variety of health services and/or clinics that service a diverse population of consumers. However, routine collection of these measures across health services has proven to be difficult, particularly at follow-up assessment, resulting in low completion rates, ranging typically from 1 to 45% [18–22]. This is problematic because low completion rates reduce the accuracy of PROM data and may provide a biased indication of healthcare performance [8, 9].

In 2019, Peninsula Health implemented the routine collection of the European Quality of Life Five Dimension instrument (EQ-5D-5L) at two of its multidisciplinary community health services for the purpose of measuring changes in health-related quality of life, and informing provider-patient decisions and healthcare interventions [23]. The EQ-5D-5L is a generic measure of health-related quality of life that provides an indication of general health status across five health domains (mobility, self-care, usual activities, pain, and anxiety/depression) as well as a Visual Analogue Scale (EQ-VAS) rating of overall health between 0 and 100, where 100 indicates best possible health [24, 25]. The EQ-5D-5L was chosen for routine collection at these services due to its demonstrable validity and reliability across a diversity of health conditions [26, 27], and its potential to support value-based healthcare [23, 28–30]. Administering the EQ-5D-5L on admission and discharge, these multidisciplinary services achieved follow-up completion rates of 73% demonstrating feasibility and utility of this measure [23]. Being able to expand the use of an existing measure that met our overarching requirements is likely to support broader scale-up implementation of the EQ-5D-5L at Peninsula Health [31].

To achieve high PROM completion rates across an entire healthcare organisation it is recommended that an evidence-based systematic approach is used [32]. Such an approach should include engagement with the clinicians and consumers who will be using PROMs to ensure that their needs and preferences are considered with reference to the use and collection of these data [32–34]. However, there are few examples of engagement of stakeholders during the pre-implementation phase (i.e., planning) when deciding which PROM(s) to collect.

Consumers are rarely involved in the process of selecting an appropriate PROM for implementation across health services [22]. Instead, PROMs are typically selected based on their measurement properties, [19, 21, 35] expert opinion via consultation with clinicians, [21, 36, 37] utility as an outcome measure and/or the current or historical use of PROMs [20]. While these criteria are important to consider, engaging consumers in the process provides complementary information that can guide selection of a PROM that consumers find acceptable for use in their care [34, 38]. This is vital because successful implementation (i.e., high completion rates) of a PROM is dependent on its acceptability to both those who administer it (i.e., clinicians) and those who complete it (i.e., consumers) [39].

Acceptability has been defined as ‘a multi-faceted construct that reflects the extent to which people delivering or receiving a healthcare intervention consider it to be appropriate, based on anticipated or experienced cognitive and emotional responses to the intervention’ [39]. It consists of seven component constructs which may be considered by the individual when determining acceptability of a PROM, including: affective attitude (i.e., how an individual feels about the PROM); burden (i.e., perceived amount of effort required to complete the PROM); ethicality (i.e., does the PROM fit within individual’s value system); intervention coherence (i.e., individual’s understanding of the PROM and how it works); opportunity costs (i.e., benefits or values that the individual must sacrifice to engage with the PROM); perceived effectiveness (i.e., individual’s perception that the PROM captures relevant aspects of their health); and self-efficacy (i.e., individual’s confidence that they can understand and complete the PROM) [39]. While previous implementation research has explored consumer acceptability of kidney disease specific PROMs for use in routine symptom assessment [22, 40], the acceptability of generic PROMs, such as the EQ-5D-5L, for routine use in care has not been established. Assessing consumer acceptability of the EQ-5D-5L in the multidisciplinary health services at Peninsula Health can provide evidence-based information to support making decisions on its appropriateness, and strategy for, wider implementation across the organisation.

The primary aim of this study was to establish the acceptability of using the EQ-5D-5L in routine care from the perspective of clients receiving healthcare. The secondary aim was to determine if acceptability varied by client sub-types.

## Methods

### Study design

A cross-sectional survey design was used to explore acceptability of the EQ-5D-5L in routine care from the perspective of clients who received care within these services. The study received ethics approval from the Peninsula Health Human Research Ethics Committee (approval no. LNR/78268/PH-2021-274394).

### Setting

This study is part of a broader body of work undertaken in the establishment of the National Centre for Healthy Ageing, Healthy Ageing Data Platform, a collaboration between Monash University and Peninsula Health in Victoria, Australia [41]. Central to the Data Platform is the implementation and integration of a system for routine collection of PROMs across an entire healthcare organisation with a view to expanding collection to the entire geographic region. This PROMs data will be used for the purpose of measuring changes in health-related quality of life (i.e., measured at multiple timepoints), and informing provider–patient decisions and healthcare interventions across the entire healthcare organisation.

Peninsula Health is a publicly funded metropolitan healthcare organisation in Melbourne, Australia. It provides hospital (two acute and two sub-acute hospitals) and more than 10 community-based health services, across a range of different clinical specialties, for over 300,000 people [42].

### Overarching methodological framework

**Fig. 1:**
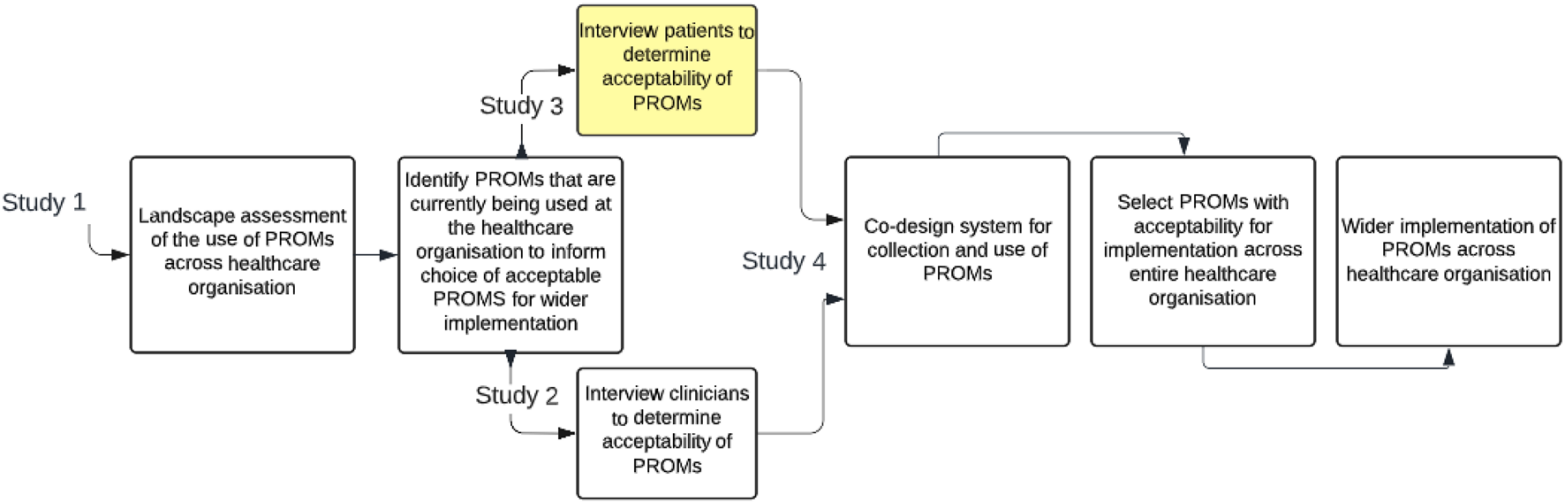
Planning implementation of routine collection of Patient Reported Outcome Measures across Peninsula Health

To expand PROMs collection across the entire organisation we proposed a program of work consisting of four studies (Fig. 1) that address the five core steps involved in planning implementation of routine collection of PROMs [32–34, 43]. These include: (1) purpose of PROM collection; (2) scope of the PROM; (3) practical considerations of PROM collection; (4) patient and therapist acceptability of the PROM; and (5) measurement properties of the PROM [32–34, 43]. Study 1 was a landscape assessment of current use of PROMs across Peninsula Health that established that the EQ-5D was the most used across all clinical specialties [43]. Based on the findings of study 1, the acceptability of the EQ-5D-5L for use in routine care was explored in studies 2 and 3 to determine whether it was suitable for wider implementation across Peninsula Health. Study 2 informed that the EQ-5D-5L was an acceptable PROM for use in routine care from the perspective of clinicians [44]. In this current study (study 3), we aimed to determine the acceptability of the EQ-5D-5L from the perspective of clients of Peninsula Health.

### Participants

Participants were adult clients (aged ≥ 18 years) who had completed the EQ-5D-5L on admission and discharge to one of two multi-disciplinary community health services. One was the community rehabilitation program; a home and centre-based program which provides short term rehabilitation, typically 2 to 12 weeks, for people with sub-acute illness and/or injury. The other was the community care program; a home-based program which provides long term care-coordination services, typically 3 to 6 months, for people with chronic and complex health conditions. Clients of these services have typically been referred from other services (e.g., such as acute hospital or sub-acute rehabilitation) within the healthcare organisation.

As part of routine care, carers (e.g., family, next of kin) complete the EQ-5D-5L on behalf of clients with cognitive (e.g., Alzheimer’s disease) or communication impairment (e.g., Aphasia). Therefore, we also recruited carers who completed the EQ-5D-5L. Clients were excluded from the study if they were: under 18 years of age; referred for palliative/end of life care; were cognitively impaired and did not have a carer to complete survey on their behalf; or were deceased.

A member of the research team (LM), who did not provide care to the clients, administered acceptability surveys via telephone after clients had completed the EQ-5D-5L on discharge from the health service. Prior to administering the survey, the research team member explained the purpose of the survey and research study; informed consent was implied if the client completed the survey. Data were collected from October 2021 to June 2022. All eligible clients who attended the participating health services during this time period were approached to participate in the study. Participants could withdraw from the study prior to their data being combined with other participants’ data. Participants who chose not to complete the EQ-5D-5L as part of their routine care were not eligible to participate in the study.

### Survey design

First, clients were provided with the experience of using the EQ-5D-5L during their routine care, by administering the PROM on admission (i.e., baseline assessment) and before discharge (i.e., follow-up assessment). Clients completed the ‘EQ-5D-5L telephone interview version’, and carers completed the ‘EQ-5D-5L interviewer administered proxy version 2’ which required the carer to report on the client’s health-related quality of life as they judged the client would report. Following completion of the EQ-5D-5L at discharge, clients’ and carers’ acceptability of the EQ-5D-5L was measured using a separate survey consisting of items that have been used in previous evaluations of client experience with PROMs across a range of health conditions [45, 46]. The eight items measured the clients’ experience of completing the EQ-5D-5L and were mapped to the component constructs of acceptability as outlined in Table 1 [39]. For example, item 6 asks ‘How much do these items help your therapist understand your current state of health?’, which can be mapped to the ‘perceived effectiveness’ component construct of acceptability. Consistent with the previous evaluations, client level of agreement with items 1 to 5 was indicated on a four-point Likert scale (‘strongly disagree’ to ‘strongly agree’), and client response to item 6 is provided on a five-point Likert scale (‘Not at all’ to ‘A lot’) [45]. Clients provided open-ended responses to items 7 and 8. Surveys took approximately 5 to 10 min to complete.

**Table 1:** Survey items

Item	Format	Component construct of acceptability
1 The items of the EQ-5D-5L were easy to understand	Likert^a^	Intervention coherence; self-efficacy
2 I found these items to be useful	Likert^a^	Perceived effectiveness
3 Answering these items improved my communication with my therapist in this department	Likert^a^	Perceived effectiveness
4 Answering these items made me feel more in control of my own care in this department	Likert^a^	Perceived effectiveness
5 I would like to continue using the EQ-5D-5L to provide information on how I am doing	Likert^a^	Burden; opportunity cost
6 How much do these items help your therapist understand your current state of health?	Likert^b^	Perceived effectiveness
7 Describe the aspects of your illness/condition that had a big impact on your health, but were not captured by the EQ-5D-5L	Open-ended	Perceived effectiveness
8 Describe the aspects of your illness/condition that changed during the course of your care	Open-ended	Perceived effectiveness

^a^Four-point scale: ‘strongly disagree’ to ‘strongly agree’

^b^Five-point scale: ‘Not at all’ to ‘A lot’

Demographic data (i.e., age, gender) and basic health information (i.e., diagnosis, co-morbidities) were extracted from client medical records.

### Analysis

Responses to the Likert scale items were analysed descriptively using proportions (%). Fischer’s exact tests were used to determine association between responses to Likert scale items and age (i.e., less than 60 years of age vs. 60 years or older), sex (i.e., male vs. female), responder (i.e., proxy vs. self-completion), referral diagnosis (i.e., respiratory vs. neurological vs. orthopaedic vs. mobility/falls vs. other) and multimorbidity (i.e., 2 or more comorbidities vs. 1 or fewer comorbidities) with α = 0.05. Statistically significant associations were further investigated via examination of cell proportions, and calculation of Cramer’s V with interpretation guided by Cohen [47, 48]. For a test with 1 degree of freedom, Cramer’s V of 0.10 is considered a small effect, 0.30 a medium effect and 0.50 a large effect [48]. Pairwise Pearson correlation coefficients were calculated to investigate correlation among item responses and were interpreted as follows: 0 to .30 negligible correlation; .30 to .50 low; .50 to .70 moderate; .70 to .90 high; and .90 to 1.0 very high [49].

Responses to open-ended items (items 7 and 8) were analysed using qualitative content analysis [50]. This involved three researchers (DAS, LM, KN) familiarising themselves with the data by reading and re-reading responses. The researchers then independently classified the text into meaning units (i.e., words and sentences related to each other through their content and context). Meaning units were then condensed, labelled as a code and sorted into categories through discussion between the three researchers. The researchers re-read responses to confirm the codes/categories and ensure no new codes/categories arose. The proportion (%) of participants who reported each category was then calculated.

## Results

**Fig. 2:**
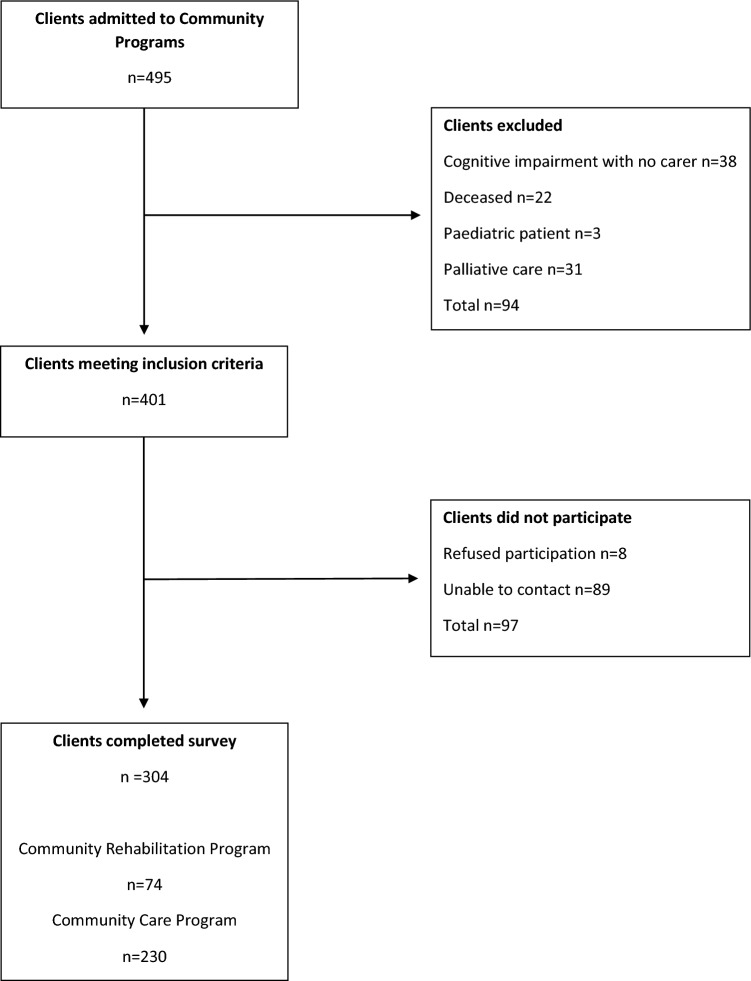
Participant flow chart

Of the 401 clients who met the inclusion criteria, 304 (76%) completed the acceptability survey (Fig. 2). The number of participants based on diagnosis classification were: mobility/fall n = 45 (15%); neurological n = 60 (20%); orthopaedic n = 64 (21%); respiratory disease n = 34 (11%); and other n = 101 (33%). A detailed summary of diagnoses is provided in Supplementary File 1.

Participants were mostly female (n = 191, 63%) with an average age of 70 years (SD 16). Two-hundred and two (66%) participants had a co-morbidity, with the most common co-morbidities being respiratory disease (n = 57, 19%), diabetes (n = 53, 17%), mental health disorder (n = 47, 16%), cancer (n = 43, 14%), cerebrovascular disease (n = 36, 12%) and cardiac disease (n = 31, 10%). Twenty-six (9%) surveys were completed by proxy.

While participant characteristics were mostly similar to non-responder characteristics, non-responders were more likely to have an ‘other’ diagnosis (responders n = 101, 33% vs. non-responders n = 48, 49%). Participant and non-responder characteristics are reported in Table 2.

**Table 2:** Participant characteristics

Characteristic	Responders	Non-responders
	n (%)	n (%)
	n = 304	n = 97
Age* (years)	70 (16)	68 (19)
Sex		
Female	191 (63)	55 (57)
Male	113 (37)	42 (43)
Referral diagnosis		
Mobility/fall	45 (15)	12 (12)
Neurological	60 (20)	15 (15)
Orthopaedic	64 (21)	13 (13)
Other	101 (33)	48 (49)
Respiratory disease	34 (11)	9 (9)
Responder		
Proxy	26 (9)	–
Self-completion	278 (91)	–
≥ 2 Comorbidities	85 (28)	21 (22)
Comorbidities		
Cancer	43 (14)	11 (11)
Cardiac disease	31 (10)	11 (11)
Cerebrovascular disease	36 (12)	10 (10)
COVID-19	10 (3)	2 (2)
Dementia	16 (5)	4 (4)
Diabetes	53 (17)	9 (9)
Hepatic disease	7 (2)	2 (2)
Mental health disorder	47 (16)	20 (21)
Renal	14 (5)	5 (5)
Respiratory disease	57 (19)	14 (14)

*Mean (SD)

### Acceptability of EQ-5D-5L

**Fig. 3:**
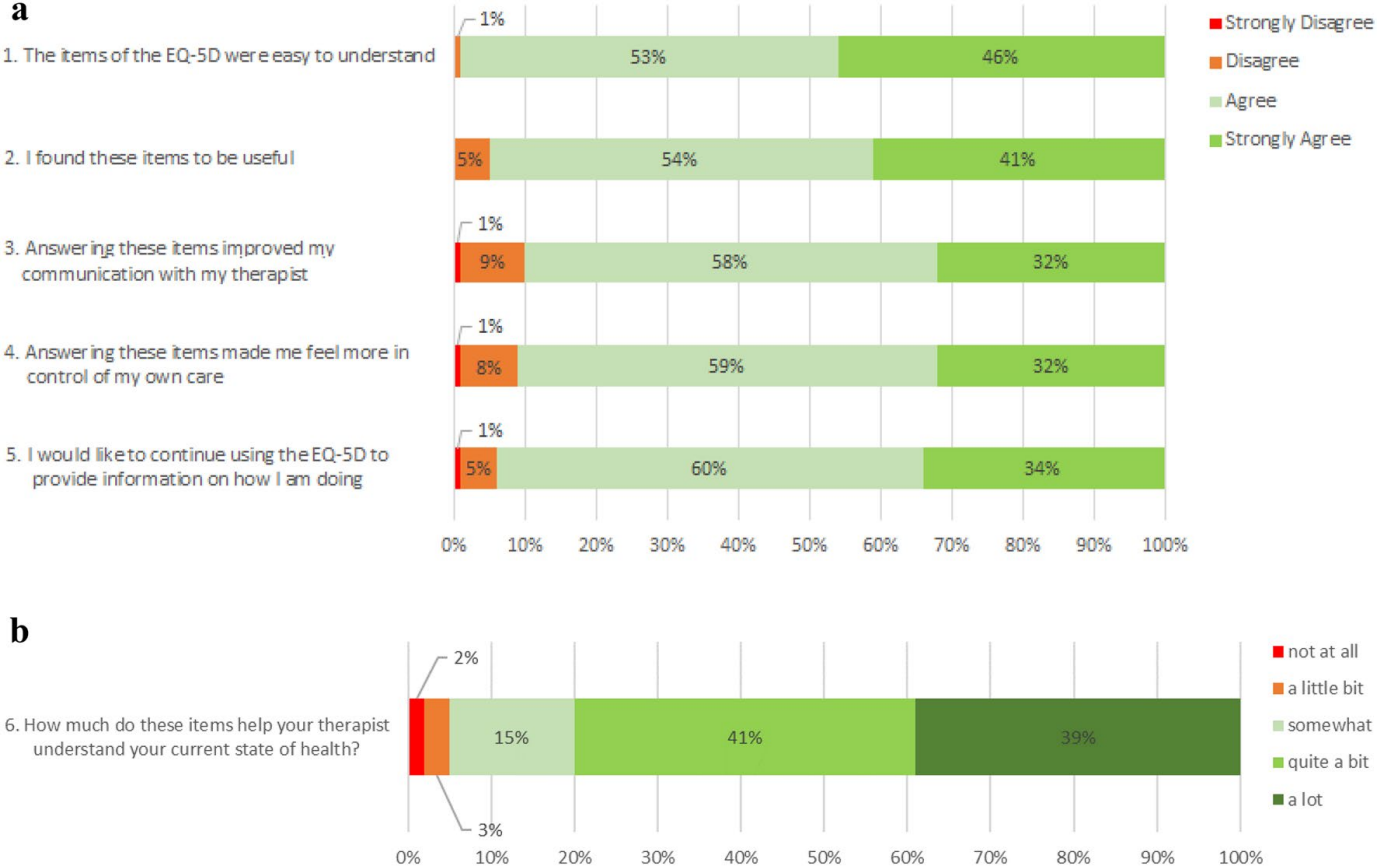
**a** Participant response distribution to items 1-5 on experience with EQ-5D-5L. **b** Participant response distribution to item 6 on experience with EQ-5D-5L

Overall, participants reported that the EQ-5D-5L was acceptable with 93% of participants (n = 285) indicating that they would like to continue using the EQ-5D-5L (Fig. 3a). The majority of participants agreed or strongly agreed that the EQ-5D-5L items were easy to understand (n = 301, 99%) and useful (n = 289, 95%). Most participants also agreed or strongly agreed that answering the EQ-5D-5L items improved communication with their therapist (n = 275, 90%) and made them feel more in control of their own care (n = 276, 91%). In response to the question ‘How much items help your therapist understand your current state of health?’, 15% (n = 45) responded ‘somewhat’, 41% (n = 124) responded ‘quite a bit’ and 39% (n = 120) responded ‘a lot’.

**Fig. 4:**
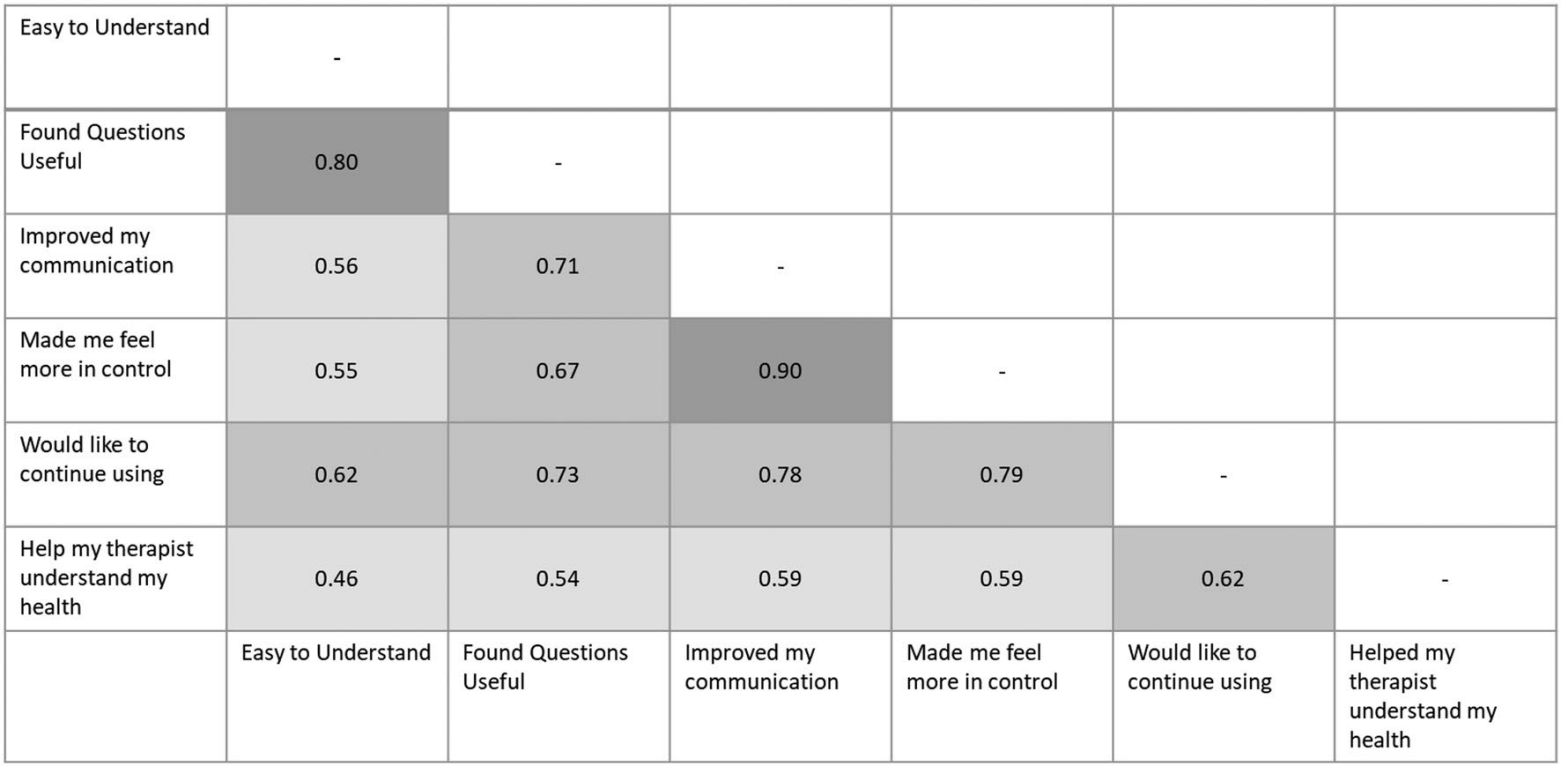
Correlation between item responses

Three items were strongly correlated with intent to continue using the EQ-5D-5L: ‘I found these items to be useful’ (r = .73); ‘improved my communication with my therapist’ (r = .78); and ‘feel more in control of my own care’ (r = .79) (Fig. 4). There was also a very high correlation (r = .90) between responses to items ‘improved my communication with my therapist’ and ‘feel more in control of my own care’.

Age was the only factor associated with acceptability (Table 3, 4). Participants aged ≥ 60 years were less likely to ‘strongly agree’ that completing the EQ-5D-5L improved their communication with their therapist (χ^2^ = 13.81; df = 3; p = .003) and increased control of their own care (χ^2^ = 11.17; df = 3; p = .011) compared to those < 60 years. Those aged ≥ 60 years were also less likely to agree that they wanted to continue using the EQ-5D-5L (χ^2^ = 10.46; df = 3; p = .015), and less likely to report believing that completing the EQ-5D-5L helped their therapist understand their health state (χ^2^ = 19.59; df = 4; p < .001). On further investigation via Cramer’s V, these associations are small in magnitude according to Cohen’s guidelines [48].

**Table 3:** Association between acceptability of the EQ-5D-5L and age, sex, responder and comorbidities

Likert response	Age			Sex			Responder			Comorbidities			
	< 60	≥ 60	P	Female	Male	P	Proxy	Self	P	< 2	≥ 2	P	
Total	77 (25)	227 (75)		191 (63)	113 (27)		26 (9)	178 (91)		219 (72)	85 (28)		304 (100)
Item 1. Easy to understand													
Strongly disagree	0 (0)	0 (0)	.155	0 (0)	0 (0)	.614	0 (0)	0 (0)	.784	0 (0)	0 (0)	.367	0 (0)
Disagree	0 (0)	3 (100)		2 (67)	1 (33)		0 (0)	3 (100)		3 (100)	0 (0)		3 (1)
Agree	35 (22)	126 (78)		97 (60)	64 (40)		15 (9)	146 (91)		112 (70)	49 (30)		161 (53)
Strongly agree	42 (30)	98 (70)		92 (66)	48 (34)		11 (8)	129 (92)		104 (74)	36 (26)		140 (46)
Item 2. Found items useful													
Strongly disagree	0 (0)	0 (0)	.213	0 (0)	0 (0)	.153	0 (0)	0 (0)	.476	0 (0)	0 (0)	.567	0 (0)
Disagree	4 (27)	11 (73)		6 (40)	9 (60)		2 (13)	13 (87)		10 (67)	5 (33)		15 (5)
Agree	35 (21)	129 (79)		103 (63)	61 (27)		16 (10)	148 (90)		115 (70)	49 (30)		164 (54)
Strongly agree	38 (30)	87 (70)		82 (66)	43 (34)		8 (6)	117 (94)		94 (75)	31 (25)		125 (41)
Item 3. Improved communication													
Strongly disagree	0 (0)	3 (100)	**.003**^a^	0 (0)	3 (100)	.100	0 (0)	3 (100)	.632	1 (33)	2 (67)	.518	3 (1)
Disagree	5 (19)	21 (81)		16 (62)	10 (38)		2 (8)	24 (92)		19 (73)	7 (27)		26 (9)
Agree	34 (19)	142 (81)		108 (61)	68 (39)		18 (10)	158 (90)		127 (72)	49 (28)		176 (58)
Strongly agree	38 (38)	61 (62)		67 (68)	32 (32)		6 (6)	93 (94)		72 (73)	27 (27)		99 (33)
Item 4. Feel more in control of care													
Strongly disagree	1 (25)	3 (75)	**.011**^**b**^	1 (25)	3 (75)	.367	0 (0)	4 (100)	.680	2 (50)	2 (50)	.796	4 (1)
Disagree	4 (17)	20 (83)		15 (63)	9 (37)		2 (8)	22 (92)		17 (71)	7 (29)		24 (8)
Agree	36 (20)	144 (80)		111 (62)	69 (38)		18 (10)	162 (90)		130 (72)	50 (38)		180 (59)
Strongly agree	36 (38)	60 (62)		64 (67)	32 (33)		6 (6)	90 (94)		70 (73)	26 (27)		96 (32)
Item 5. Like to continue using													
Strongly disagree	0 (0)	2 (100)	**.015**^**c**^	0 (0)	2 (100)	.050	0 (0)	2 (100)	.907	0 (0)	2 (100)	.124	2 (1)
Disagree	3 (18)	14 (82)		7 (41)	10 (59)		2 (12)	15 (88)		11 (65)	6 (35)		17 (6)
Agree	36 (20)	144 (80)		113 (63)	67 (37)		16 (9)	164 (91)		132 (73)	48 (27)		180 (59)
Strongly agree	38 (36)	67 (64)		34 (32)	71 (68)		8 (8)	97 (92)		29 (28)	76 (72)		105 (35)
Item 6. Help therapist understand													
Not at all	1 (17)	5 (83)	**.001**^**d**^	2 (33)	4 (67)	.273	0 (0)	6 (100)	.599	4 (67)	2 (33)	.241	6 (2)
A little bit	2 (20)	8 (80)		4 (40)	6 (60)		1 (10)	9 (90)		4 (40)	6 (60)		10 (3)
Somewhat	4 (9)	40 (91)		29 (66)	15 (34)		5 (11)	39 (89)		32 (73)	12 (27)		44 (15)
Quite a bit	24 (19)	100 (81)		77 (62)	47 (38)		13 (10)	111 (90)		92 (74)	32 (26)		124 (41)
A lot	46 (38)	74 (62)		79 (66)	41 (34)		7 (6)	113 (94)		87 (73)	33 (27)		120 (40)

Bold value indicates statistical significance (*P* < 0.05)

^a^Cramer’s V = 0.21

^b^Cramer’s V = 0.19

^c^Cramer’s V = 0.19

^d^Cramer’s V = 0.25

**Table 4:** Association between acceptability of the EQ-5D-5L and referral diagnosis

Likert response	Referral Diagnosis						
	Mobility/fall	Neurological	Orthopaedic	Other	Respiratory	P	
Total	45 (15)	60 (20)	64 (21)	101 (33)	34 (11)		304 (100)
Item 1. Easy to understand							
Strongly disagree	0 (0)	0 (0)	0 (0)	0 (0)	0 (0)	.421	0 (0)
Disagree	1 (33)	0 (0)	1 (33)	1 (33)	0 (0)		3 (1)
Agree	19 (12)	27 (17)	34 (21)	61 (38)	20 (12)		161 (53)
Strongly agree	25 (18)	33 (24)	29 (21)	39 (28)	14 (10)		140 (46)
Item 2. Found items useful							
Strongly disagree	0 (0)	0 (0)	0 (0)	0 (0)	0 (0)	.437	0 (0)
Disagree	3 (20)	1 (7)	3 (20)	7 (47)	1 (7)		15 (5)
Agree	18 (11)	32 (20)	38 (23)	58 (35)	18 (11)		164 (54)
Strongly agree	24 (19)	27 (22)	23 (18)	36 (29)	15 (12)		125 (41)
Item 3. Improved communication							
Strongly disagree	1 (33)	0 (0)	1 (33)	1 (33)	0 (0)	.368	3 (1)
Disagree	10 (38)	2 (8)	9 (35)	10 (38)	1 (4)		26 (9)
Agree	27 (15)	36 (20)	38 (22)	56 (32)	19 (11)		176 (58)
Strongly agree	34 (34)	22 (22)	15 (15)	34 (34)	14 (14)		99 (33)
Item 4. Feel more in control of care							
Strongly disagree	0 (0)	0 (0)	2 (50)	2 (50)	0 (0)	.546	4 (1)
Disagree	4 (17)	2 (8)	8 (33)	9 (38)	1 (4)		24 (8)
Agree	25 (14)	37 (21)	39 (22)	59 (33)	20 (11)		180 (59)
Strongly agree	16 (17)	21 (22)	15 (16)	31 (32)	13 (14)		96 (32)
Item 5. Like to continue using							
Strongly disagree	0 (0)	0 (0)	1 (50)	1 (50)	0 (0)	.589	2 (1)
Disagree	4 (23)	2 (12)	3 (18)	7 (41)	1 (6)		17 (6)
Agree	21 (12)	35 (19)	44 (24)	61 (34)	19 (11)		180 (59)
Strongly agree	20 (19)	23 (13)	16 (9)	32 (31)	14 (13)		105 (35)
Item 6. Help therapist understand health							
Not at all	2 (33)	0 (0)	2 (33)	2 (33)	0 (0)	.636	6 (2)
A little bit	2 (20)	2 (20)	1 (10)	5 (50)	0 (0)		10 (3)
Somewhat	3 (7)	6 (14)	12 (27)	17 (39)	6 (14)		44 (15)
Quite a bit	22 (18)	24 (19)	27 (22)	36 (29)	15 (12)		124 (41)
A lot	16 (13)	28 (23)	22 (18)	41 (34)	13 (11)		120 (40)

Thirty-eight categories, relating to aspects of health, were created from qualitative content analysis of open-ended responses. The definition and an example quote are provided for each category in Supplementary File 2. The most commonly reported aspects of health that clients noted the EQ-5D-5L didn’t capture were fatigue (n = 38, 13%), balance/falls (n = 34, 11%), respiratory symptoms (n = 30, 10%), physical strength (n = 28, 9%), cognition (n = 26, 9%), and sleep (n = 26, 9%). (Table 5). Four aspects of health commonly identified by participants as missing from the EQ-5D-5L are in fact captured by the EQ-5D-5L. These were pain unrelated to reason for referral (n = 30, 10%), social activities (n = 16, 5%), driving (n = 7, 2%), and return to work (n = 7, 2%).

**Table 5:** Consumer reported aspects of illness/condition not captured by the EQ-5D-5L

Aspect	n (%)
Fatigue	38 (13)
Balance/falls	34 (11)
Pain unrelated to reason for referral*	30 (10)
Respiratory symptoms	30 (10)
Physical strength	28 (9)
Cognition	26 (9)
Sleep	26 (9)
Weight	25 (8)
Range of motion	20 (7)
Sensations	19 (6)
Medication side effects	18 (6)
Physical support aids	18 (6)
Social activities*	16 (5)
Wounds	16 (5)
Nutrition/diet	15 (5)
Vestibular	15 (5)
Emotions other than anxiety/depression	14 (5)
Appetite	12 (4)
Gastrointestinal	12 (4)
Financial	9 (3)
Incontinence	8 (3)
Self-efficacy	8 (3)
Vision	8 (3)
Driving*	7 (2)
Relationships	7 (2)
Return to work*	7 (2)
Speech	6 (2)
Hearing	5 (2)
Swelling	5 (2)
Alcohol/drugs	4 (1)
Other neurological symptoms	4 (1)

Total respondents n = 304

*Captured by EQ-5D-5L

The most common aspects of health reported by consumers to have changed during the course of care included usual activities (n = 73, 24%), mobility (n = 60, 20%), pain/discomfort (n = 36, 12%), use of physical support aids (n = 19, 6%), emotions other than anxiety/depression (n = 16, 5%), physical strength (n = 15, 5%), and respiratory symptoms (n = 15, 5%) (Table 6).

**Table 6:** Consumer reported aspects of illness/condition that changed during the course of rehabilitation

Aspect	n (%)
Usual activities*	73 (24)
Driving	13 (4)
Return to work	12 (4)
Social activities	6 (2)
Mobility*	60 (20)
Pain/discomfort*	36 (12)
Pain unrelated to reason for referral	6 (2)
Physical support aids	19 (6)
Emotions other than anxiety/depression	16 (5)
Physical strength	15 (5)
Respiratory symptoms	15 (5)
Wounds	13 (4)
Nutrition/diet	12 (4)
Anxiety/depression*	11 (4)
Self-care*	11 (4)
Balance/falls	10 (3)
Cognition	10 (3)
Range of motion	10 (3)
Weight	10 (3)
Self-efficacy	6 (2)
Vestibular	6 (2)
Fatigue	5 (2)
Speech	5 (2)
Appetite	4 (1)
Gastrointestinal	4 (1)
Alcohol/drugs	3 (1)
Incontinence	3 (1)
Sleep	3 (1)
Medication side effects	2 (1)
Sensations	2 (1)
Vision	2 (1)
Hearing	2 (1)
Financial	1 (< 1)
Other neurological symptoms	1 (< 1)
Relationships	1 (< 1)
Swallowing	1 (< 1)

Total respondents n = 304

*Captured by EQ-5D-5L

## Discussion

Our results indicate that routine use of the EQ-5D-5L was acceptable for a diverse population of healthcare consumers. The majority of consumers indicated that they were happy to continue using the EQ-5D-5L as they found it was easy to use, improved communication with their therapist, and made them feel more in control of their own care. Importantly, they identified that the EQ-5D-5L measures the most common aspects of health that changed over the course of their care, including participation in usual activities, mobility, and pain. However, the EQ-5D-5L did not capture all aspects of health that were relevant to their illness/condition, such as fatigue, respiratory symptoms, and cognition. It may also be less acceptable for people over the age of 60.

Previous evaluations of consumer experience with the EQ-5D-5L have reported that it is easy for consumers to understand and use during the course of their care [51–53]. Our findings expand on this by highlighting that consumers, with a diversity of health conditions, see benefit in completing the EQ-5D-5L, particularly for enhancing communication with their therapists and control of their own care. Further, the high correlation between these items suggests that these two benefits may be linked; specifically, the EQ-5D-5L likely facilitates greater perception of control via enhanced communication between therapist and patient. It is likely that these benefits are related to the way in which the therapist uses the EQ-5D-5L in building therapeutic relationships, rather than from the administration of the tool on its own [54, 55]. For example, therapists who probe consumer responses on the EQ-5D-5L to better understand their health state and those who take steps to address the problems raised by consumers will be more likely to foster a meaningful therapeutic relationship than therapists who ignore consumer responses [54]. Our results are encouraging and indicate that the EQ-5D-5L can positively influence consumers’ communication with their therapist and control of their own care and that this in turn influences consumers desire to continue using the PROM.

Our results also compare favourably to evaluations of consumer experience with other generic PROMs, such as the Patient-Reported Outcomes Measurement Information System (PROMIS) [45, 56]. While healthcare consumers with rheumatological and neurological disease have reported mostly positive experiences with the PROMIS, a higher proportion of consumers in our study reported improved communication (90% vs. 78%), control of own health (91% vs. 70–71%), and intent to continue using the EQ-5D-5L (93% vs. 79–81%) [45, 56]. However, in these evaluations the PROMIS was administered in combination with condition specific PROMs, and differences should be interpreted within this context.

The majority of participants reported that the EQ-5D-5L was acceptable for use in their routine care, reporting high levels of acceptability across the component constructs of acceptability (≥ 90% agreement on each item). This finding indicates that the EQ-5D-5L meets the needs of many patients within our healthcare network, an important consideration when implementing routine collection of PROMs spanning a number of program areas, and therefore is an appropriate PROM for routine collection [32]. Previous research has indicated that choosing a PROM which is user friendly is most important when considering the needs of patients [32]. However, our findings show that patients’ perceptions of the usefulness of the items and their impact on their care (i.e., communication and control) are strongly correlated with whether they intend to continue using the PROM. This highlights the importance of establishing patient opinions on the content of PROMs when selecting a PROM for routine collection.

Although the majority of participants reported that the EQ-5D-5L was acceptable for use in their routine care, those aged 60 years or older reported lower levels of acceptability (‘agree’ rather than ‘strongly agree’) within the ‘burden’, ‘opportunity cost’ and ‘perceived effectiveness’ component constructs of acceptability. To our knowledge, globally this is the first study to find a negative association between older age and acceptability of the EQ-5D-5L, which may be explained by the limited capacity of the EQ-5D-5L to capture all aspects of health-related quality of life that are relevant to older people (i.e., perceived effectiveness) [57]. The risk of complex disease significantly increases with age and negatively impacts on health-related quality of life [58, 59]. Therefore, it may be less likely that the EQ-5D-5L will capture all aspects of health that are affected for those with complex disease (i.e., limited perceived effectiveness). Further research is required to understand why the EQ-5D-5L is less acceptable in those aged 60 years or older.

It is not uncommon for older people to experience difficulty interpreting and understanding items on the EQ-5D-5L [60]. Previous evaluations of the EQ-5D-5L have found that older people have particular difficulty with the usual activities and pain/discomfort dimensions [57, 61]. Specifically, prior studies report that older people perceive the usual activities dimension as being only one activity rather than multiple activities and interpret pain/discomfort as either only pain or only discomfort [57, 61]. Some participants in our study had similar difficulties with these dimensions; not identifying that social activities, return to work and driving are captured by the usual activities dimension, and also not understanding that the pain/discomfort dimension can refer to pain/discomfort experienced from a source other than that of the reason for referral to the health service. Given the recurring difficulties that individuals have interpreting these items, the EuroQoL Group may consider revising the standardised script for the EQ-5D. For example, the usual activities item provides examples that the respondent might consider, including work, study, housework, family and leisure activities. Consideration might be given to including driving and social activities. Similarly, clarification that ‘pain/discomfort’ can relate to any pain or discomfort that is experienced may be considered to improve interpretation of this item for respondents who are receiving treatment for a pain disorder of a particular body part/region.

While the EQ-5D-5L captured most of the commonly reported aspects of health that consumers identified had changed throughout the course of their care, it did not capture all aspects relevant to consumers. This finding is consistent with a previous evaluation of the EQ-5D-5L in a younger cohort with chronic diseases which also found that fatigue was the most commonly reported aspect of health not captured [46]. Similarly, adults with asthma reported that the EQ-5D-5L did not adequately capture respiratory symptoms, which is consistent with the experience of our older cohort of consumers [61]. Unique to our cohort was that consumers identified falls and balance, cognition, and sleep disturbances as aspects of health not captured by the EQ-5D-5L. These findings may be explained by the relative older age of our consumers compared to previous evaluations [46, 60], and highlights the importance of evaluating acceptability in the intended population of consumers, as results may vary between geographic locations and settings.

Given its overall level of acceptability and capacity to capture the aspects of health that matter to consumers, the EQ-5D-5L appears to be an acceptable measure of health-related quality of life with good potential for wider implementation in our healthcare setting. However, there were issues with acceptability that could be addressed to improve the likelihood of successful implementation (i.e., high completion rates). First, the addition of items such as fatigue and cognition may improve consumer acceptability by better capturing relevant aspects of health. Notably, fatigue and cognition were only reported as ‘not captured’ by 13% and 9% of consumers, respectively. However, improving consumer acceptability of the EQ-5D-5L in as little as 10% of consumers could have positive implications for completion rates and ensuring that data is representative of the consumer population [8, 9]. The addition of fatigue and cognition items to the EQ-5D-5L has also previously been trialled and found to improve the psychometric properties of the tool in patients with chronic disease and stroke [62, 63]. Further, disease-specific PROMs or items could be used to complement the EQ-5D-5L and better capture the experience of health for clients with complex disease. However, for routine collection, consideration needs to be given to responder burden as this can impact on response rates [21]. Specifically, the EQ-5D-5L typically takes less than 5 minutes to complete, which is seen as an acceptable feature of the tool [53]; adding additional items to better capture aspects of health would increase the time taken to complete the EQ-5D-5L which may lead to higher responder burden, less consumer acceptability and lower response rates. Last, knowledge that the EQ-5D-5L has potential to enhance (1) the therapeutic relationship and (2) consumers’ perceptions of control of their own care could be used to convince clinicians of the clinical utility of PROMs, a known barrier to routine collection of PROMs [8]. Support and training for clinicians on how to use PROMs in clinical care to identify and address the problems reported by consumers may also enhance consumers’ experience of care across the healthcare organisation which, in turn, should increase consumer response rates [32, 54, 55, 64].

Our study has several strengths and limitations. We achieved a response rate of 73% in a large sample with representation from a wide variety of diagnoses, predominantly chronic in nature. Consistent with an older population of healthcare consumers, participants in our study presented with numerous comorbidities. This diversity in comorbidities made it difficult to determine how diagnosis and/or co-morbidities influence acceptability. We used pre-established questions to determine consumer acceptability based on literature review and previously used in similar populations across numerous studies [45, 46, 56, 65]. A limitation of our study is that the psychometric properties (i.e., reliability and validity) of these items have not been formally tested. However, by mapping these items to the Sekhon et al. acceptability framework [39] we were able to ensure that the relevant constructs of acceptability were addressed. A further limitation of our study is the generalisability of our findings to other healthcare settings, which may not reflect the acceptability of the EQ-5D-5L in different settings and geographical locations. While we captured the views of a diverse population, we may not have identified acceptability issues relevant to specific diseases [57]. Further studies are required to examine acceptability of the EQ-5D-5L within disease-specific cohorts. Importantly, we were able to capture the perspectives of consumers with cognitive impairment through proxy surveys. We acknowledge that there are limitations in using proxies to complete PROMs on behalf of consumers, as proxy report of consumers’ health may differ from consumers’ self-report of their own health [66]. However, proxy completion is important for consumers who cannot complete PROMs, such as those with advanced cognitive impairment, to ensure that consumers such as these are not excluded from the benefits of using PROMs [67, 68]. Also, while our results did not indicate that proxy experiences of using the EQ-5D-5L differed to those of consumers, this could be further explored in a larger sample.

## Conclusions

Routine use of the EQ-5D-5L in clinical care was mostly acceptable across a diverse population of consumers. While this suggests that it is an appropriate generic PROM for routine collection in our healthcare setting, there are features of the EQ-5D-5L which are less acceptable to consumers which could be addressed to facilitate uptake. In particular, the addition of items relating to fatigue and cognition may improve the acceptability of the EQ-5D-5L for consumers. Also, the capacity of PROMs to improve communication between therapist and consumers highlights that PROMs data needs to be readily accessible to therapists. Knowledge gained from this study demonstrates the importance of consumer engagement and will be critical in facilitating the successful implementation of routine collection of the EQ-5D-5L across a healthcare organisation.

### Supplementary Information

Below is the link to the electronic supplementary material.

Supplementary file1 (PDF 840 kb)

## Data Availability

Data is available from the authors upon reasonable request.
