# Supplementary material for: Healthcare consumer acceptability of routine use of the EQ-5D-5L in clinical care: a cross-sectional survey
Source: Qual Life Res. 2024 Feb 7;33(5):1307–21. doi: 10.1007/s11136-024-03598-z (PMC11045645; doi:10.1007/s11136-024-03598-z)
Supplement: Supplementary file 1 — Supplementary file1 (PDF 840 kb) [file 11136_2024_3598_MOESM1_ESM.pdf]

Supplementary File 1: Detailed summary of diagnoses

Referral diagnosis	n
**Mobility/Fall**	
Decline in mobility	7
Fall	38
**Neurological**	
Aphasia	1
Carpal tunnel	1
Cerebellar ataxia	2
Cervical myelopathy	1
Chiari malformation	1
Dementia	8
Dysarthria	1
Epilepsy	1
Functional neurological disorder	4
Multiple sclerosis	3
Parkinson's disease	3
Seizures	3
Spinal cord injury	1
Stroke	18
Vertigo (central)	12
**Orthopaedic**	
Elective arthroplasty – Lower limb	22
Joint dislocation	4
Lower limb fracture	22
Mandibular fracture	1
Spinal fusion	2
Upper limb fracture	10
Vertebral fracture	3
**Other**	
Acute myeloid leukaemia	1
Alcohol and drug addiction	2
Anaemia	1
Angina	3
Anxiety	2
Aspiration	1
Back pain	8
Bladder cancer	1
Breast cancer	1
Cardiac bypass	1
Cardiac failure	4
Cellulitis	1
Cirrhosis	1
Cold agglutinin disease	1

Colectomy - Colostomy formation	2
Colectomy small bowel resection	2
Constipation	1
Delirium	1
Depression	2
Diabetes	7
Dysphagia	1
Fibromyalgia	1
Financial distress	2
Hyperemesis	1
Hypoglycaemia	1
Infected sebaceous cyst	1
Inguinal Hernia	1
Laparotomy	1
Laryngospasm	1
Liver failure	1
Liver resection	2
Lower limb amputation	3
Macular degeneration	1
Malnutrition	3
Melanoma	1
Nephrectomy	1
Oesophageal strictures	1
Osteoarthritis	4
Osteomyelitis	1
Ovarian cancer	1
Peri-rectal abscess	1
Polymyalgia rheumatica	1
Primary sclerosing cholangitis	1
Proctitis	1
Prostate cancer	4
Renal cancer	1
Renal failure	5
Shoulder pain	2
Syncope	3
Urinary retention	1
Urinary tract infection	1
Wound	8
**Respiratory disease**	
Asthma	1
Chronic cough	1
COPD	18
COVID-19	7
Lobectomy	1
Pneumonia	2
Pulmonary embolism	1
Pulmonary fibrosis	1

Sleep apnoea	1
Tuberculosis	1

Supplementary File 2: Categories of aspects of health

**Category**	**Definition**	**Example quote (i.e. meaning unit)**
Alcohol/Drugs	Misuse of alcohol or drugs including addiction.	*“I am a lifelong alcoholic with terminal liver failure”*
Anxiety/Depression	Feelings of anxiousness and/or depression that have improved or worsened over time, inclusive of related psychological disorders such as suicidal ideation and post-traumatic stress.	*“Depression is getting worse and worse. Everything is going down my depression is bad.”*
Appetite	Changes in desire or interest in food.	*“My appetite is not what it was because of the bowel problems.”*
Balance/Falls	Loss of balance, feelings of unsteadiness, occurrence of falls, fear of falls.	*“I have no balance. My legs get stuck in one spot and I fall flat on my face.”*
Cognition	Common signs of cognitive impairment including memory loss or decline, forgetfulness, confusion, losing a train of thought or specific words, and difficulties with flow of conversations.	*“My memory loss. I can't find all the words I am looking for sometimes.”*
Driving (usual activities)	Inability to drive or reduced capacity to drive.	*“Can't drive at all anymore.”*
Emotions other than anxiety/depression	Negative emotional or psychological states other than anxiety or depression; mood changes, anger, stress, helplessness, frustration, paranoia.	*“I am frustrated that I can't do what I used to do.”*
Fatigue	Tiredness, exhaustion, drowsiness, reduced stamina and energy levels.	*“Fatigue. I get tired all the time doing the smallest of things like walking to the letterbox. I can't get through shopping without tiredness.”*
Financial	Financial barriers to affordable healthcare, adequate food, and housing. Strain on finances as a result of health condition/s, applications for pensions or disability funding.	*“Finances, I couldn't pay bills due to my stroke and fall. Have a healthcare card to help survive.”*
Gastrointestinal	Symptoms of gastrointestinal upset from illness or medications or issues relating to surgery of the gastrointestinal system.	*“I had my stoma reversed recently, it consumes my life every day with how my bowels are working. I can't get constipated or get diarrhoea. Never settled with the stomach.”*
Hearing	Impairment or loss of/changes in hearing in one or both ears, with or without the use of aids.	*“My hearing is getting worse, I can barely hear on the phone.”*

Incontinence	Unintentional urination; needing to use continence aids; difficulties with toileting.	*"Wearing nappies now because I can't get to the toilet quick."*
Medication side effects	Reporting specifically on the side effect/s of their medication	*"Side effects of medications, Endone makes me so tired. On antibiotics for the rest of my life, which gives me reflux."*
Mobility	Walking ability, transferring, and/or the need for mobility aids.	*"Couldn't walk to start with, and now getting around with a walker."*
Nutrition/Diet	Medically indicated modifications to dietary intake, maintenance of nutritionally balanced diets, barriers to meal preparation and cooking, presence of food intolerances, and the use of liquid supplements for weight maintenance and nutritional support.	*"Meals. My diet is a big problem, have needed meals on wheels. I couldn't eat at all, food fatigue."*
Other neurological symptoms	Involuntary movements- shaking, tremors, seizures.	*"Tremors in my hand. Jerking a lot, like involuntary movements."*
Pain/Discomfort	Any pain or discomfort of any body part, including acute and chronic pain.	*"Pain is so much better since doing physiotherapy exercises".*
Pain unrelated to reason for referral (pain/discomfort)	Pain that is unrelated to the primary reason for referral.	*"Pain down the leg all day. I have had chronic pain anyway, every day, for years."*
Physical support aids	Any aid that is used to physically support mobility, showering, sleep or specific body parts.	*"I use my wheelchair when I want to go out anywhere, and my walker around the house."*
Range of motion	How far a body part can move or stretch, including touching the floor (bending), squatting and reaching**.**	*"Can't squat down or bend, putting my arms away from my side I cannot do."*
Relationships	Personal relationships with spouses, family members or friends, and having limited access to support.	*"Relationship problems also, marriage problems. Didn't capture those. My marriage breakdown influenced all my health problems and downward spiral."*
Respiratory symptoms	Symptoms of pulmonary diseases (e.g. shortness of breath, coughing) and associated treatments including oxygen therapy.	"My breathing, I am short of breath because of the COPD. Still on the oxygen."
Return to work (usual activities)	Capacity or ability to return to work duties.	*"The impact on my work too. My lawn mowing business. My son had to take over."*
Self-Care	Capacity to wash and dress; toileting, personal care of dressings/wounds.	*"I can't cook, clean or shower by myself; that is not getting better."*

Self-efficacy	Self-confidence to perform tasks effectively, such as walking, resuming normal activities, and self-care.	*"The stroke I had. Total loss of confidence, especially with my walking and showering alone."*
Sensations	Experiences of paraesthesia due to neuropathy (nerve damage), characterized by perceptions of numbness (loss of sensation), paralysis (loss of movement/sensation), burning sensations, or temporary paraesthesia (pins and needles). *False perceptions or "hallucinations" of objects or events involving senses* of sight, sound, smell, touch and taste. Sudden feelings of warmth in the upper body region (hot flushes).	*There's a sensation of warmth on the right-hand side of the body. Pins and needles come and go but much better than constantly there like they used to be.*
Sleep	Quality, timing and amount of sleep.	*"Not sleeping as well anymore, very irregular. I wake up during the night."*
Social activities (Usual activities)	Ability to socialise with others; participation in social activities.	*"I am back to bingo, and going down to the veterans club"*
Speech	Ability to speak, including loss of voice, slurred or stuttered speech, or problems with speaking.	*"His speech is really affected, getting worse. It's all slurry and mumbly."*
Strength (physical)	Muscle strength/weakness; general strength/weakness; ability to perform fine/gross motor tasks.	*"Muscle strength. I used to be fit and strong. It's so much less now."*
Swallowing	Ability to swallow food or drinks.	*"It's not bad, I use more of the drinks rather than solid food because my throat is really closed up now. The dietician at Frankston helped me a lot with the swallowing, I have the milks."*
Swelling	Swelling or lymphoedema in any part of the body.	*"My swelling is my main concern, I was in hospital for 5 days recently whilst they drained it all."*
Usual Activities	Ability to perform usual activities e.g. work, study, housework, family or leisure activities.	*"Now I am going out for dinner with my family."*
Vestibular	Symptoms of vestibular (inner ear and brain) disorders including vertigo and dizziness.	*"I am always giddy and have dizziness."*
Vision	Ability to see; deterioration of eyesight causing impaired vision or blindness.	*"I am blind in my left eye, and have only half sight on the right."*
Weight	Ability to attain/maintain a healthy body weight; undesirable weight gain or weight loss; inability to reduce body weight as a function of illness, medications, lack of mobility, mental health issues or loss of appetite.	*"His weight loss, he lost a lot of weight in hospital, he had no appetite, so sick."*

Wounds	Any damage to the skin and/or flesh of the body tissue, inclusive of ulcers, sores, lesions, blisters and abscesses; wound care.	*“My wounds. The skin graft didn't take, and I have to have my wound dressing changed every day, wish that was only twice a week as it's annoying to do that so often.”*
